# A Review of Subsequence Time Series Clustering

**DOI:** 10.1155/2014/312521

**Published:** 2014-07-21

**Authors:** Seyedjamal Zolhavarieh, Saeed Aghabozorgi, Ying Wah Teh

**Affiliations:** Department of Information Systems, Faculty of Computer Science and Information Technology, University of Malaya (UM), 50603 Kuala Lumpur, Malaysia

## Abstract

Clustering of subsequence time series remains an open issue in time series clustering. Subsequence time series clustering is used in different fields, such as e-commerce, outlier detection, speech recognition, biological systems, DNA recognition, and text mining. One of the useful fields in the domain of subsequence time series clustering is pattern recognition. To improve this field, a sequence of time series data is used. This paper reviews some definitions and backgrounds related to subsequence time series clustering. The categorization of the literature reviews is divided into three groups: preproof, interproof, and postproof period. Moreover, various state-of-the-art approaches in performing subsequence time series clustering are discussed under each of the following categories. The strengths and weaknesses of the employed methods are evaluated as potential issues for future studies.

## 1. Introduction 

One of the primary tasks of data mining is clustering, whose function groups similar objects into a cluster. Clustering is the most prevalent task of statistical data analysis in various aspects. In cluster analysis, most similar data objects are discovered on the basis of some criteria for comparisons. Clustering aims to increase the efficiency of similarity among members in a cluster [1].

In the clustering domain, Han et al. [2] propose clustering method categorizations to arrange various static data. Data are considered static if their feature values do not change with time or do not change negligibly. They divide clustering methods into five major categories, namely, partitioning, hierarchical, density-based, grid-based, and model-based methods.

In contrast to static data, time series values change with time [3]. A series of data points in similar time spaces is called time series, which is usually expressed by line charts. Information and data mining research has played an important role in the pattern-mining domain through the huge collection of time series data. The pattern discovery problem addressed by unsupervised learning is known as time series clustering [4, 5]. One of the most challengeable clustering issues in the time series data mining community [6–11] is time series clustering [12–18]. Furthermore, the high-speed growth of computer and Internet technology increases the amount of data in different fields, such as e-commerce [19], outlier detection [20, 21], speech recognition [22], biological systems, DNA optimization [23], and text mining. Among these different fields, the use of subsequence time series data is important for improving time series mining.

In time series clustering, subsequence time series clustering is proposed to group interesting subsequence time series data in the same cluster. Subsequence time series clustering is used for discovering structures or patterns in time series data.

Subsequence time series clustering leads to many interesting data, including sequential patterns, motifs, periodic patterns, partially ordered patterns, and approximate biological sequence patterns. With approximately 10 years of active research on subsequence time series mining by data mining, machine learning, statistical data analysis, and Bioinformatics research, we believe that a systematic introduction and comprehensive overview of state-of-the-art technology in this area is warranted.

Many algorithms have used time series clustering [15, 16]. A previous study in 1998 proposes the main reference technique for single time series clustering, which is used for the subroutine rule discovery of time series [24]. In this study, the *k*-means clustering algorithm is applied to extract subsequence clusters. The problem of this method is that it shows only sine waves and has an independent output compared with the input. To solve this problem, most studies have tried to find improved algorithms for subsequence time series clustering from 1998 to 2003 [4, 24–26].

Lin et al. [27] prove the claim of meaningless results in 2003 and explain that all previous claims are false because they have the same results and are unacceptable [14]. Thus, researchers have tried to find a solution to this issue and answer the question of why it is meaningless (from 2005 to 2011) [13, 14, 28–30]. This issue has remained unsolved.

From 2011 to 2013, three main papers have proposed solutions for the problem of meaningless results of time series clustering and explained how to obtain meaningful time series clusters [31–33]. Continually, most of the papers claim that they reach appropriate results with different ways.

The rest of this paper is organized as follows.  Section 2 clarifies the main definitions and background of subsequence time series clustering.  Section 3 includes the evolution of subsequence time series clustering according to papers on the subject.  Section 4 provides the discussions, comparisons, strengths, and weaknesses. Finally, Section 5 concludes the paper.

## 2. Background and Definitions 

In this section, we provide the definitions and background knowledge used in this work.

### 2.1. Time Series Definitions

**Definition 1 .**

**Figure 1:**
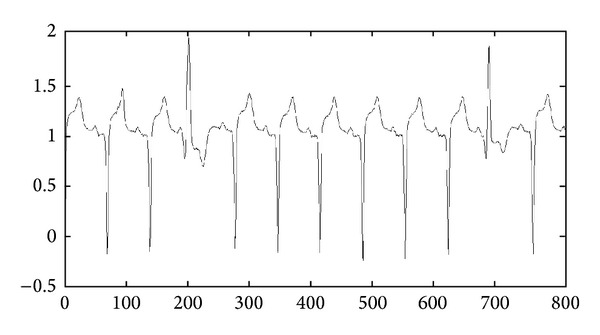
A sample of time series data.

A time series *T* of size *m* is an ordered sequence of real-value data, where *T* = (*t*_1_, *t*_2_ … *t*_*m*_) [33].  Figure 1 shows a sample of time series data.

**Definition 2 .**

A subsequence of length *n* of time series *T* is *T*_*i*,*n*_ = (*t*_*i*_, *t*_*i*+1_,…, *t*_*i*+*n*−1_), where 1 ≤ *i* ≤ *m* − *n* + 1 [33]. A subsequence is an arranged sequence of data that omits some elements without changing the order of the remaining elements [34].

### 2.2. Taxonomy of Time Series Clustering

**Figure 2:**
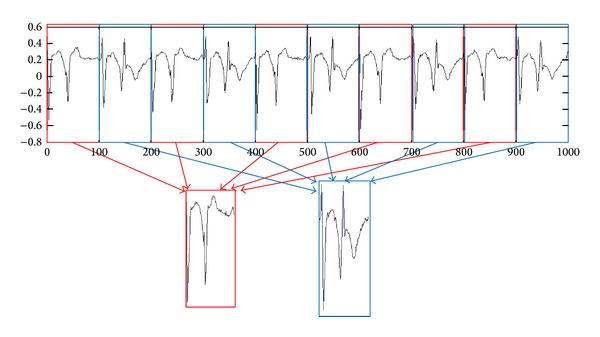
A sample of subsequence time series clustering.

**Figure 3:**
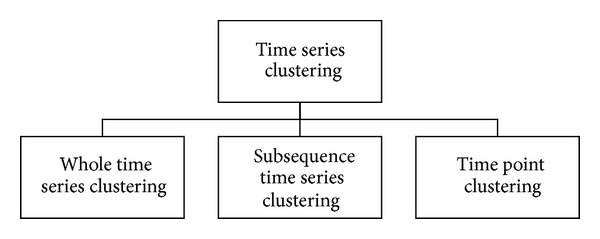
Time series clustering taxonomy.

In reviewing literature, one can conclude that most works related to clustering time series are classified into three categories: whole time series clustering, subsequence time series clustering, and time point clustering (Figure 3). The first two categories are mentioned in 2005. Whole time series clustering is the clustering of a set of individual time series with respect to their similarity. Here, clustering means the conventional (usually) clustering of discrete objects, which are time series [35, 36]. Subsequence time series clustering involves the clustering of a set of subsequences of a time series extracted via a sliding window, that is, the clustering of segments from a single long time series.  Figure 2 illustrates the subsequence clustering of time series data.

Another category of clustering is time point clustering [37–39], which is the clustering of time points on the basis of a combination of their temporal proximity and the similarity of their corresponding values. This approach is similar to time series segmentation. However, time point clustering is different from segmentation in the sense that all points do not need to be assigned to the cluster; that is, some of points are considered noise.

Subsequence clustering is performed on a single time series [14]. Time point clustering is also applied to a single time series and is similar to time series segmentation. That is, the objective of time point clustering is to find clusters of the time point instead of clusters of time series data. In the next section, subsequence time series clustering and its concepts are explained.

### 2.3. Subsequence Time Series Clustering

**Figure 4:**
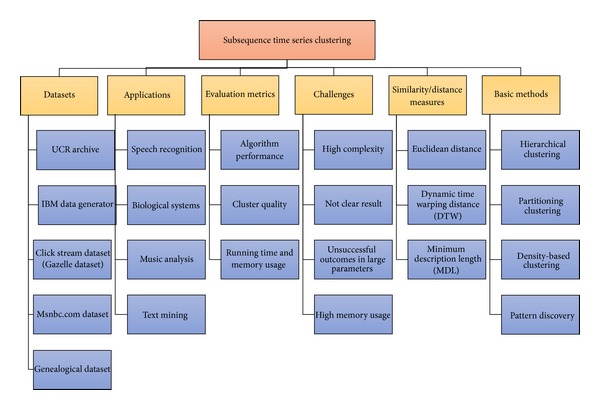
The general skeleton of subsequence time series clustering.

This paper aims to review the main concepts of subsequence time series clustering step by step.  Figure 4 elucidates the general skeleton of this clustering with some features and subfeatures covered in this paper which are utilized in most of the related work. Additionally, in the end of this part, related algorithms which have been applied in subsequence time series clustering are explained. It is important to notice that the skeleton of this paper is extracted from some works which exactly applied subsequence time series clustering; hence, it may omit some minor features.

#### 2.3.1. Basic Methods

In subsequence time series clustering, an important issue is how to employ methods for categorizing a huge amount of time series data and how they can produce a meaningful result. Some major methods, such as hierarchical clustering, *k*-means, and pattern discovery, are described briefly as follows.

*(1) Hierarchical Clustering*. One other general clustering algorithm is hierarchical clustering, which has a powerful visualization compared with other clustering approaches [40]. Hierarchical clustering creates a nested hierarchy of related groups of objects regarding a pairwise distance matrix of the objects. One of the strengths of this method is generality; that is, the user does not need to provide any parameter, such as the number of clusters. However, the application of this method is limited to small datasets because of its quadratic computational complexity [27]. The following outlines the basic hierarchical clustering algorithm.

1. The distance between all objects is calculated. The results are stored in a distance matrix.
2. Search through the distance matrix for the two most similar clusters/objects.
3. The two clusters/objects are joined to produce a cluster with at least two objects.
4. The matrix is updated by calculating the distances between this new cluster and all other clusters.
5. Step 2 is repeated until all cases are in one cluster.

Hierarchical methods are divided into two types: agglomerative and divisive. Agglomerative methods have a bottom-up structure; thus, each data object stays in one cluster and then merges with other clusters until a large cluster forms. This task continues while all clusters create the main root cluster. The structure of divisive methods is the opposite; that is, a top-down structure is applied. The cluster splits into small clusters. By merging this process with other clustering techniques, we can increase the quality of hierarchical clustering. Nevill-Manning and Witten [41] use the SEQUITUR algorithm to abstract subsequences as a hierarchical method for subsequence clustering. Kumar et al. [5] in 2006 propose an adaptive WaveSim transform on the basis of a hierarchical tree-based approach to improve subsequence time series clustering.

*(2) Partitioning Clustering.* Given a set of *n* unlabeled data tuples, a partitioning method constructs *k* partitions of the data, where each partition illustrates a cluster containing at least one object and *k* ≤ *n*. The partition is crisp if each object belongs to exactly one cluster, or fuzzy if one object is allowed to be in more than one cluster to a different degree. Two renowned heuristic methods for crisp partitions are the *k*-means algorithm, where each cluster is represented by the mean value of the objects in the cluster and the *k*-medoids algorithm, where each cluster is represented by the most centrally located object in a cluster. Two counterparts for fuzzy partitions are the fuzzy *c*-means algorithm and the fuzzy *c*-medoids algorithm. These heuristic algorithms apply well for discovering spherical-shaped clusters and small to medium data sets. To discover clusters with nonspherical or other complex shapes, specially designed algorithms such as Gustafson-Kessel and adaptive fuzzy clustering algorithms or density-based methods to be explained in the sequel are required. Most genetic clustering methodsimplement the spirit of partitioning methods, especially the *k*-means algorithm, the *k*-medoids algorithm, and the fuzzy *c*-means algorithm [42].

*(3) Density-Based Clustering*. The idea of density-based methods such as DBSCAN is to continue growing a cluster as long as the density (number of objects or data points) in the “neighborhood” exceeds some threshold. More than producing a cluster, OPTICS calculates an augmented cluster ordering for automatic and interactive cluster analysis. The ordering contains information that is equivalent to density-based clustering obtained from a wide range of parameter settings [43, 44]. Denton [45] uses this type of clustering for her work.

*(4) Pattern Discovery*. An interesting function of time series clustering is pattern discovery, which involves two major fields: frequent [46] and surprising patterns [47]. These methods are also known as motif discovery [48, 49] and anomaly [50, 51] or discord detection [52], respectively.

Pattern discovery is a significant task in data mining [53, 54]. In 2003, Ma and Perkins [55] develop a support vector regression- (SVR-) based algorithm that detects online novelties. This algorithm applies the pattern discovery method to cluster data regarding temporal sequences. In 2005, Chan and Mahoney [50] proposed an approach to determine anomalies online by using the Gecko algorithm. This method generates a sequence of minimal bounding boxes with the training trajectories. For discovering time series patterns, distance-based clustering is commonly used [24, 25, 56].

#### 2.3.2. Similarity/Distance Measures

An important clustering job is determining the similarity between two data. These data come in different forms, including raw values of equal or unequal length, vectors of feature-value pairs, and transition matrices.

*(1) Euclidean Distance*. Assuming that *x*_*i*_ and *v*_*j*_ are *P*-dimensional vectors, the Euclidean distance can be calculated as follows [3]:

(1) $$\begin{array}{c} d_{E}=\sqrt{\overset{p}{\underset{k=1}{\sum }}{\left(x_{ik}-\;v_{jk}\right)}^{2}}. \end{array}$$

*(2) Dynamic Time Warping Distance*. Dynamic time warping (DTW) generalizes classical algorithms to compare discrete sequences to continuous value sequences [3]. For time series *Q* = *q*_1_, *q*_2_ … *q*_*i*_ … *q*_*n*_ and *R* = *r*_1_, *r*_2_ … *r*_*j*_ … *r*_*m*_, DTW align the two series to minimize differences. To this end, an *n* × *m* Matrix, where the (*i*, *j*) element of the matrix covers the distance *d*(*q*_*i*_, *r*_*j*_) between points *q*_*i*_ and *r*_*j*_. In this matrix, the Euclidean distance is typically measured. A warping path *W* = *w*_1_, *w*_2_ … *w*_*k*_,…, *w*_*k*_, where max⁡⁡(*m*, *n*) ≤ *k* ≤ *m* + *n* − 1, is a set of matrix elements that satisfies three constraints: boundary condition, continuity, and monotonicity. To meet the boundary condition constraints, the warping path must start and finish in matrix corner cells that are diagonally opposite; that is, *w*_1_ = (1, 1) and *w*_*K*_ = (*m*, *n*). The continuity constraint restricts the allowable number of steps to the adjacent cells, and the monotonicity constraint forces the monotonic spacing of points on the warping path in time. A warping path that displays the minimum distance between the two time series is of interest and is expressed as follows:

(2) $$\begin{array}{c} d_{\text{DTW}\;}=\min\frac{\sum_{K=1}^{K}w_{k}}{K}. \end{array}$$

Dynamic programming efficiently determines this path by evaluating the below recurrence, which defines the cumulative distance as the sum of the distance of the current element and the minimum cumulative distance of the adjacent elements [3]:

(3) dcum(i,j)=d(qi,rj) +min⁡⁡{dcum(i−1,j−1),     dcum(i−1,j),dcum(i,j−1)}sp.

Oates [25] uses this measure in 1999 to identify distinctive subsequences. This measure has also been used by Rakthanmanon et al. [57] in 2013 to address significant time series data.

*(3) Short Time Series Distance*. Short time series (STS) distance is the squared of the gradient distance between two time series data [58]. Mathematically, the STS distance between two time series *x*_*i*_ and *v*_*j*_ is defined as

(4) $$\begin{array}{c} d_{\text{STS}}=\;\sqrt{\overset{p}{\underset{k=1}{\sum }}{\left(\frac{v_{j(k+1)}-v_{jk}}{t_{\left(k+1\right)}-t_{k}}-\frac{x_{i(k+1)}-x_{ik}}{t_{\left(k+1\right)}t_{k}}\right)}^{2}}. \end{array}$$

In this formula *t*_*k*_ is the time point for data points *x*_*ik*_ and *v*_*jk*_, and *z* standardization is used for deleting the effect of scale.

*(4) Minimum Description Length*. The minimum description length (MDL) supplies a criterion for model selection regardless of complexity without the restrictive assumption that the data generate a sample from a “true” distribution. Algorithms such as PRESEE have applied MDL as a base [59].

#### 2.3.3. Challenges

The clustering of subsequence time series is hindered by the following issues with respect to algorithm behavior.

*(1) High Memory Usage.* In the clustering of subsequence time series, the memory consumed by linear spaces and large clustering data is problematic. A prevalent weakness that is commonly observed in studies on subsequence time series is increased memory usage, which reduces clustering efficiency [32, 41, 60–63].

*(2) Unsuccessful Outcomes with Large Parameters.* Algorithms must occasionally analyze many parameters, thus severely affecting the clustering of subsequence time series and rendering the examined parameters meaningless. This problem is common in the second period (interproof period) of subsequence time series clustering, which is explained in the next section (Section 3.2) [4, 5, 61–63].

*(3) Unclear Result*. The results of most papers in the second period of subsequence time series analysis are unclear. All of the clusters evaluated by using various algorithms display similar results with no remarkable difference. Consequently, the researchers considered the clusters to be meaningless [5, 14, 27, 32, 64].

*(4) High Complexity.* The uncertainty of the solution eliminates the dismissal property, and high complexity is generated when the results do not match the objective of the clustering of large time series. The time complexity will be minimal if ignored.

#### 2.3.4. Evaluation Metrics

In this section, we clarify some of the criteria that directly affect the evaluation of algorithms for subsequence time series clustering.

*(1) Cluster Quality (Accuracy)*. To evaluate clustering quality, studies use cross entropy, which is expressed as follows:

(5) $$\begin{array}{c} \text{Cross}\;\text{entropy}=\;\overset{k}{\underset{j=1\;}{\sum }}\left(\frac{n_{j}}{\left|\text{SDB}\right|}\right)\left(-\;\overset{m}{\underset{i=1}{\sum }}p_{ij}\log\left(p_{ij}\right)\right), \end{array}$$

where *K* is the prespecified number of clusters; *n*_*j*_ represents the number of sequences in the *j*th cluster; *m* is the number of natural classes in the sequence database; *p*_*ij*_ is the probability of randomly drawing sequences from the *j*th cluster that belongs to class *i*; SDB is the sequence database. This equation assesses algorithm accuracy.

*(2) J*-*Measure*. Regarding measure rule of algorithms, *J*-measure use apply rule-ranking. It is defined as

(6) J(BT;A) =p(A)∗(p(BT ∣ A)log⁡⁡(p(BTA)p(BT))       +(1−p(BT ∣ A))log⁡⁡(1−p(BT ∣ A)1−p(BT))),

where, in the context of sequence rules, *p*(*A*) is the probability of symbol *A* occurring at a random location in the sequence, *p*(*B*_*T*_) is the probability of at least one *B* occurring in a randomly chosen window of duration *t*, and *p*(*B*_*T*_∣*A*) is the probability of at least one *B* occurring in a randomly chosen window of duration *T* given that the window is immediately preceded by an *A*. Intuitively, the first term in the *J*-measure, namely *p*(*A*), is a bias towards rules which occur more frequently [24, 65].

*(3) Normalized Mutual Information (NMI)*. NMI is one of the significant comparing measures for evaluating clustering results of algorithms. It can help researchers to assess algorithm performance and analyze their improvements. It is specified as follows.

Let *C*_*T*_ and *C*_*E*_ be the set of true class labels and the set of cluster labels calculated by a clustering algorithm, respectively.

Then, NMI between *C*_*T*_ and *C*_*E*_ is

(7) $$\begin{array}{c} \text{NMI}\left(C_{T},C_{E}\right)=\frac{H\left(C_{T}\right)+H\left(C_{E}\right)}{H\left(C_{T},C_{E}\right)}\left(=1+\frac{I\left(C_{T};C_{E}\right)}{H\left(C_{T},C_{E}\right)}\right), \end{array}$$

where *H*(*P*), *H*(*P*, *Q*), and *I*(*P*; *Q*) represent entropy, joint entropy, and mutual information with respect to random variables *P* and *Q*. When *C*_*T*_ and *C*_*E*_ are independent from one another, NMI (*C*_*T*_, *C*_*E*_) = 1 because *H*(*C*_*T*_, *C*_*E*_) = *H*(*C*_*T*_) + *H*(*C*_*E*_) should be satisfied. The larger the NMI (*C*_*T*_, *C*_*E*_) is, the more accurate the clustering results are [66].

*(4) Algorithm Performance.* Algorithm performance is typically evaluated by the following measurements.

1. *Test Environment and Datasets.* Previous studies have shown that mining closed sequential patterns can lead to more concise sets of results than mining all sequential patterns. To assess the algorithm performance, we consider the use of datasets because their features affect this performance.
2. *Scalability Test.* In the experiments, we replicate the dataset from 1 time to 20 times. The constraint and the threshold of minimum relative support are set. Both the runtime of and space usage by the algorithm increased linearly when the number of input sequences increased, and this observation implies that the base size algorithm is scalable. By testing the scalability of the algorithm and by determining the relationship between this scalability and the other criteria, we can evaluate the algorithm performance.

*(5) Runtime and Memory Usage*. By measuring runtime and memory usage, we can compare algorithms with respect to the time and memory consumed. Furthermore, we can calculate the minimal support threshold in the algorithm. The runtime and memory usage of an algorithm increase exponentially as the minimal threshold decreases.

#### 2.3.5. Applications

In this section, we describe some of the major applications of subsequence time series clustering.

*(1) Speech Recognition.* Subsequence time series clustering is typically applied in speech processing. Thus, this study examines each stage of a speech-recognition system. Speech-recognition engines can be improved by matching a detected word to a known word by using an online pattern recognition [22, 33].

*(2) Biological Systems.* Time series expression experiments have been used to investigate numerous biological systems. Many bioinformatics problems, including heartbeat-related illnesses (electrocardiography (ECG) data analysis) and analyses of human wellbeing and genes, can be improved by this method [33, 67].

*(3) Music Analysis.* In music analysis, determining the underlying natural structures of sequences is an interesting challenge from demonstration programming to code optimization. This problem can be addressed by using the online pattern recognition method.

*(4) Text Mining.* Subsequence time series clustering can efficiently illustrate text mining projects on a discrete analogue of time series, such as English texts [32].

#### 2.3.6. Datasets

In this section, some useful datasets that have been applied in subsequence time series clustering are clarified.

*(1) Archive of the University of California at Riverside.* The library of Special Collections and University Archives at UCR house rare books, manuscripts, archival materials, photographs, videotapes, broadsides, and other media formats that cover a wide range of special subject areas. In the domain of the subsequence time series clustering, most studies use this archive as a reference source in implementing their algorithms. The most prevalent datasets used in subsequence time series clustering are ECG, cylinder-bell-funnel (CBF), speech data, music, and video data.

(*2) IBM Data Generator*. This generator is a synthetic data generator that prepares open source data by using IBM Quest version and C#. These data can generate transactions, sequences, and taxonomies.

*(3) Clickstream Dataset (Gazelle Dataset).* This dataset contains 59,601 sequences of clickstream data in FrameCommerce, with 497 distinct items. The sequences are 2.42 items long on average with a standard deviation of 3.22. However, 318 of these sequences are long and are composed of more than 20 items.

*(4) Msnbc.com Dataset*. This clickstream dataset has 989,818 sequences obtained from the UCI repository. After removing the shortest sequences, 31,790 sequences remain. In this dataset, 17 distinct items are located in the domain of webpage category. The average number of item sets per sequence is 13.33, and the average number of varied items per sequence is 5.33.

*(5) Genealogical Dataset*. This dataset contains different sources for different domains that are relevant to research interests. To extract information systematically from primary sources, many events are inevitably recorded; however, these events cannot be fitted to the main family trees either because the key linking information is missing or because the people concerned are not related to the researchers. Thus, the recorded information on these datasets may not be directly relevant to our personal interests.

#### 2.3.7. Related Algorithms

In this section, some of the related algorithms which have been used in subsequence time series clustering are discussed.

*(1) K* *-Means*. A common algorithm in subsequence time series clustering is *k*-means [68]. The basic intuition behind the *k*-means algorithm (and a general class of clustering algorithms known as iterative refinement algorithms) is provided as follows [27].

1. Determine a value for *k*.
2. Initialize the *k* cluster centers (randomly, if necessary).
3. Select the class memberships of *N* objects by assigning these objects to the nearest cluster center.
4. Reestimate the *k* cluster centers by assuming that the memberships found above are correct.
5. If none of the *N* objects changes the membership in the last iteration, exit the algorithm; otherwise, return to Step 3.

*(2) SEQUITUR.* SEQUITUR shapes a grammar from a sequence based on repeated phrases in that sequence. Each repetition produces a rule in the grammar, and the repeated subsequence is changed from a nonterminal symbol, creating a more brief representation of the overall sequence. The algorithm forms and maintains the grammar. Then it provides a hierarchical structure for the sequence [41, 69].

*(3) Rule Finding.* Rule discovery method works on discovering local relationships from tile series, in the spirit of association rules, sequential patterns, or episode rules [24].

*(4) L*-*Sequences*. In the discovery of variable-length distinctive subsequences we need to identify a set of fixed-length subsequences that capture patterns generated by *R*. This is completed by randomly sampling sequences of length *L*, called *L*- sequences, from the source. We need to construct a *n*-by-*n* similarity matrix and cluster of *L*-sequences and then for each of the *k* resulting clusters, where *L* is a user-specified parameter, we should choose a prototype by discovering the sequence that minimizes the average distance to all other sequences in the cluster [25].

*(5) PERUSE*. The goal of PERUSE is to find the patterns used most frequently to produce segments of the time series data that it obtains as input. Note that PERUSE must search over two spaces to discover candidates with high scores [4].

*(6) Cluster-Buster*. The cluster-buster algorithm consists of three steps [26].

1. Apply one pass of the forward algorithm to obtain the log likelihood score *s*[*i*] for each subsequence beginning at nucleotide 1 and ending at nucleotide *i*.
2. For each of these subsequences, we need to observe the end-point *b* to be reliable, but the start-point could be unreliable. Apply the backward algorithm beginning at band continuing to refine the optimal start point.
3. Ignore subsequences that overlap higher scoring subsequences with a greedy algorithm.

*(7) EM.* In order to evaluate the parameters of a Gaussian mixture model in the domain of time series data expectation maximization (EM) will be utilized. It is applied as an alternative and complemented to empirical orthogonal function (EOF) analysis. The resulting weights, associating time points with component distributions, are used to distinguish physical regimes. This method can use accurate explanation of the variability in the basic EOF analysis [27].

*(8) SOMS.* In the context of artificial neural network (ANN), one of the unsupervised algorithms which are used for maps is self-organizing map (SOM). A self-organizing map includes nodes or neurons. There are differences between this algorithm and the other artificial neural networks. The structure in this algorithm is not based on neighborhood function to preserve the topological properties of the input space. However, each node in this algorithm has a weight vector of the same dimension as the input data vectors and a position in the map space. Each node arranges in a two-dimensional regular spacing in a hexagonal or rectangular grid. This algorithm has clarified a mapping from a higher to lower dimensional input map space. The goal of this algorithm is finding the nearest node in order to place a vector from data space onto the map. This similarity (nearest) can be applied by measuring weight vector from distance metric [27].

*(9) Motif Discovery.* Motif discovery aims to find the closest subsequence from a given cluster center. The time series motif includes two most similar subsequences in the given time series [31, 70, 71].

*(10) Continuous Random-Walk Noise.* The algorithm uses a coordinate transformation on the feature space that produces a uniform noise threshold for all valid input sequences. Evaluation was based on a new measure that tests the success and validity of discovering cluster members from noise. According to the new evaluation measure, the quality of the results is enhanced by more than two orders of magnitude on some data sets compared with *k*-means [60].

*(11) Adaptive WaveSim Transform.* WaveSim transform is an approach for producing wavelet transform like coefficients by exploiting a conventional similarity measure between the function *f*(*t*) and the wavelet. WaveSim transform measures temporal data at multiple resolutions and it also generates flexibility to the user for adopting his own similarity measure between the basis function and the basis function spanning the *f*(*t*) segment [5].

*(12) RD Algorithm*. The radial distribution (RD) function can be normalized based on the total number of points. It describes how density varies as a function of distance from a reference particle. Hence there is no need to explicitly measure constant factors, and they will be removed from cluster [13].

*(13) CONTOUR.* CONTOUR evaluates a set of summarization subsequences, which is a brief representation of the original sequence database and maintains much structural information and can be applied to the input sequences with a high clustering quality [62].

*(14) Repetitive Gapped Subsequence*. Feature vector of navigation patterns is constructed with repetitive support of subsequence. The patterns repeat frequently in some sequences while the other infrequent could be discriminating features for clustering. This character motivates the construction of new feature vectors [72].

*(15) Online Motif Discovery.* There is significant research on discovering motifs in static offline databases. However, the demands of online data bases express the necessity of generating online motifs discovery. The online motif of length *m* of a time series *x* = (*x*_1_, *x*_2_,…*x*_*t*_) is a pair of subsequences (*x*_*i*,*m*_, *x*_*j*,*m*_) for 1 ≤ *i* < *i* + *m* ≤ *j* ≤ *t* − *m* + 1 such that distance (*x*_*i*,*m*_, *x*_*j*,*m*_) is the smallest among all such pairs [70].

*(16) MDL-Based Discovery.* Minimum description length supplies a criterion for the selection of models, regardless of their complexity, without the restrictive assumption that the data form a sample from a “true” distribution. Recently, some of the algorithms such as PRESEE use MDL as base of algorithm [32, 59, 63].

*(17) K*-*Best Motif Discovery.* A parameter-free motif discovery algorithm called kBMD finds *k*-best motif in any time series sequence without the need of any parameters. The algorithm returns a small set of motifs, which are ranked by a scoring function [73].

*(18) Grammar Induction.* The grammar induction in time series can discover repeated patterns without prior knowledge of their lengths. The motifs discovered by the visualization system are variable lengths in two ways. Not only can the intermotif subsequences have variable lengths, but the intramotif subsequences also are not restricted to have identical length [74].

*(19) Selective Sequence Time Series.* A new STS clustering framework for time series data called selective subsequence time series (SSTS) clustering generates meaningful results by applying an idea of data encoding to only essential subsequences cluster [33].

*(20) GOAL.* God's algorithm (GOAL) is an algorithm that only keeps the mean and standard deviation using the online O (1) incremental calculations. GOAL is a lower bound on the fastest possible algorithm for either ED or DTW subsequence search with unconstrained length queries [57].

## 3. Evolution of Subsequence Time Series Clustering

In this study, we divide the subsequence time series clustering into three categories, namely, pre-, inter-, and postproving a main problem. This categorization process is based on the claim of meaningless results reported in 2003 by Lin et al. with respect to the subsequence time series clustering [27]. Studies published prior to Keogh's claim are relegated to the preproof period, whereas research related to the claimed proof and works that attempt to develop a solution are categorized under the interproof period. Studies that provide increasingly efficient solutions are grouped into the final period. We also compare articles obtained from each category in terms of the following five features: (1) problem, (2) method, (3) algorithm, (4) goal, and (5) extension. Subsequently, we specifically detail the categories and their features.

### 3.1. Preproof Period (1997 to 2003)

In this section, we assess some of the papers published in subsequence time series clustering between 1997 and 2003. During this period, researchers explain the concepts of subsequence time series clustering and some implementation guidelines. In the following paragraphs, we briefly discuss these articles.

First, Nevill-Manning and Witten [41] have established SEQUITUR, an algorithm that represents the hierarchical structure of sequence data. This algorithm is based on the concept of abstracting subsequences that occur more than once into rules and consecutively repeating this operation. The algorithm observes two constraints: every diagram in the grammar must be unique, and every rule must be applied more than once. SEQUITUR operates incrementally and is subject to a caveat regarding the register model of computation in linear space and time. This efficiency enables its application in sequences up to 40 MB long in various domains. However, the researchers do not assess the prediction accuracy of SEQUITUR and evaluate the compression performance of SEQUITUR instead. SEQUITUR is one of the best compression algorithms, particularly when a large amount of text is available [41]. The greatest limitation of SEQUITUR is its memory usage, which is linearly associated with grammar size. Approximate versions of the algorithm can be developed to partition the input and remerge the generated grammars, thus establishing an algorithm with logarithmic memory requirements.

In another study, Das et al. [24] focus on determining the rules that either relate time series patterns to other patterns in that series or link patterns in one series to those in another. They emphasize the discovery of local patterns in multivariate time series, unlike the traditional method of time series analysis that examines mostly global models. Das et al. developed adaptive techniques to determine rules regarding the above type based on time series data. These methods discrete sequences by using vector quantification methods. They first form subsequences by using the sliding window approach through the time series. These subsequences are clustered by suitably measuring time series similarity. The discretized version of the time series is generated by obtaining the cluster identifiers that correspond to the subsequences. Once the time series data are discretized, simple methods to determine rules from the sequence are applied. The empirical results obtained by this method are provided.

Oates [25] proves that the solution to sequences can be achieved in times and spaces that are approximately associated with the total length of the sequences linearly. Although this study concentrates on multivariate and real-valued time series, the applied approach covers categorical sequences. Oates terms these processes as distinctive subsequences because the identified patterns distinguish the time series under consideration from other time series generated by the same source. However, these approaches are limited to a univariate time series and are inapplicable to problems such as the single time series.

Oates [4] also proposes PERUSE, an unsupervised algorithm for detecting recurring patterns in time series. This algorithm was tested by using sensor data from a mobile robot, that is, multivariate time series that is noisy and real-valued with variable intervals between observations. The experimental results of this study show that PERUSE can discover audio data patterns that correspond to recurring words uttered in natural languages, as well as sensor data patterns of a mobile robot reflecting the qualitatively different outcomes of taking action.

The final article published in this period [26] uses the modeling approach to discover sequence regions that are more related to the statistical model of a motif cluster than to a model of “background DNA.” The motif cluster model represents random motifs that distribute uniformly across the region, and the background model consists of independent random nucleotides with probabilities that are estimated from their local abundances in the query sequence. In this study, Frith et al. [26] identify subsequences with maximal log likelihood ratios (i.e., subsequences with high log likelihood ratios do not overlap). However, the algorithm to compute these subsequences requires time that is proportional to the square of the sequence length and is not feasible for sequences longer than a few kilobytes. Frith et al. [26] have developed three solutions to this problem: Cister, Comet, and cluster-buster. Each method possesses advantageous features to address the problem, but they are inefficient by themselves.

**Figure 5:**
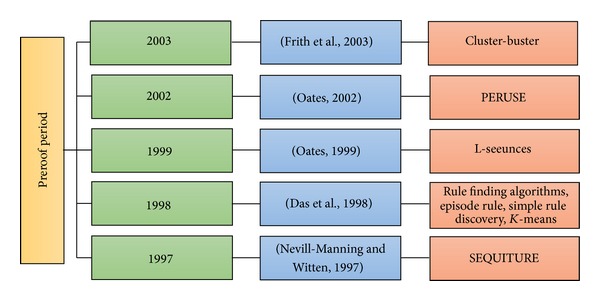
The chronology of methods in preproof period.

Table 1 illustrates the features of these articles in detail.  Figure 5 shows the methods proposed by the papers chronologically according to the year of publication and represents the slow progress of the methods in the preproof period. The development of these methods is directed toward the integration of the clustering approach into these models.

### 3.2. Interproof Period (2003 to 2010)

This period is significant in the domain of subsequence time series clustering and aims to prove the meaningless result claim by Lin et al. [27] in 2003. From 2003 to 2011, researchers have proposed solutions to this problem but with inadequate evidence. The main articles published during this period are discussed below.

The first article [27] mainly explains why the result of time series clustering is meaningless. The researchers claim the following: “the clustering of streaming time series is completely meaningless.” Clusters fetched from time series streams must follow a particular constraint, and this constraint is unlikely to be satisfied by any dataset pathologically. Thus, the clusters extracted by any clustering algorithm are essentially random. Lin et al. [27] justified their claim with a theorem, illustrative examples, and a comprehensive set of experiments on the reimplementation of previous works. Although the primary contribution of their work, which aimed to determine an apparent solution to this problem, is invalid and should not be considered, they introduced a novel method based on the concept of time series motifs, which can cluster some datasets obtained from time series streams meaningfully.

According to another study, researchers work on a never-ending learning framework for time series that tests an unbounded stream of data for a label. They demonstrate the usability of their ideas in different categories such as medicine, entomology, wildlife monitoring, and human behavior analyses. As future work, they propose to remove the few assumption/parameters in the model and also apply the idea to year-plus length streams [45].

The study by Chen [12] in 2005 is the first attempt to prove the main problem; however, this research did not successfully address the issue. Several conclusions were drawn by this work. First, sequential time series clustering can be significant. Second, accurately measuring the distances in delay space is the key to obtaining a meaningful result. He suggests that the measure of Euclidean distance adopted by most studies is flawed and presents the concept of temporal and formal similarity in delay spaces in the class of time series produced by dynamic, time invariant, and deterministic systems.

Denton [60] proposes an algorithm that incorporates a continuous random-walk noise model into kernel-density-based clustering. This algorithm not only surpasses partitioning techniques that generate unimportant and unsatisfactory results under the given quality measure, but also improves upon other density-based algorithms. The results of this study suggest that the noise elimination properties of clustering algorithms based on kernel density can be significant in the application of clustering to data preprocessing.

Keogh and Lin [14] extend the 2003 claim that the “time series subsequence clustering is meaningless” in this work. The clusters extracted from these time series must follow a certain constraint, and this constraint is unlikely to be satisfied by any dataset pathologically; therefore, the clusters extracted by any clustering algorithm are essentially random.

Goldin et al. [64] present cluster form distance, which is an alternate distance measure based on cluster shapes for subsequence time series clusters. The cluster shape is determined by the sorted list of Euclidean distances between pairwise the centroids of a set of clusters. Two algorithms are developed based on this distance measure, and these algorithms match a set of cluster centroids of the subsequence time series with the parent time series. The first algorithm creates small “fingerprints” for the sequences, whereas the second algorithm has high accuracy and correctly matches an entire dataset containing 10 sequences. Muller-Levet et al. [58] also explain why cluster shape distance matches the subsequence time series clusters to the original sequences more reliably than cluster set distance. Their work was the first to establish a strong relation between the result of the *k*-means subsequence time series clustering and its parent time series sequence despite earlier predictions of its impossibility.

Kumar et al. [5] propose methodologies to extract hidden knowledge from time series data through an unsupervised approach by using the unique WaveSim transform. This novel transform is a unique wavelet transform version and considers pattern analysis and recognition. The mining of time series data has been classified broadly into the mining of the entire series and of subsequence time series. This study proposes an approach to mine subsequence time series on the basis of a hierarchical tree by using a modified WaveSim transform called adaptive WaveSim transform.

The subsequent research of Chen [28] aims to solve meaningless problems, including clustering results for distinct time series that are indistinguishable from one another, and smoothened cluster centroids. The method proposed in this study restricts the extension of the clustering space to cover only the area containing the time series in the space of the subsequence vector. Chen reports that the approach can overcome both problems by producing significant clusters and cluster representatives that effectively correspond to (were located among) the data points in their respective clusters. Several solutions have been proposed for the dilemma of subsequence time series clustering since it was first identified in [27]; however, this method is the first to directly address both problems identified above. Hence, he establishes the term-frequency algorithm to determine useful results for time series clustering as required.

In 2007, Chen [61] indicates that sequential time series clustering is meaningful and that the problem highlighted in previous works stems from the use of the Euclidean distance metric as the distance measure in the delay-vector space. He proposes a general class of time series as a solution and presents a regime based on two types of similarities that can exist among delay vectors. A measure of distance is naturally generated as an alternative to Euclidean distance in the delay-vector space. Chen suggests that the sequential time series clustering can be significant when this alternative distance measure is applied. However, the results of the study are limited given certain barriers.

Fujimaki et al. [66] theoretically studied subsequence time series clustering from a frequency-analysis viewpoint and identified the mathematical background according to the sine wave model generation of subsequence time series clustering. This study also develops a unique theoretical analysis methodology for pattern discovery in time series data. On the basis of theoretical analysis, the clustering of phase analysis-subsequence time series (PA-STS), which aligns subsequence phases prior to clustering, is proposed. This study validates the effectiveness of PA-STS clustering when applied to time series data obtained from UCR.

Denton et al. [13] introduce a clustering algorithm that creates clusters exclusively from subsequences that occur more frequently in a dataset than expected by random chance. This algorithm partially incorporates a pattern-mining perspective into clustering. Subsequences based on such clusters need not be labeled; the subsequences in the clustering of an unrelated time series are not expected to receive labels.

Wang et al. [62] suggest a novel method for discovering the subset of useful and frequent subsequences. In this method, any existing algorithm for frequent sequence mining is used to obtain the complete set of frequent subsequences. A subset of interesting subsequences can then be identified. However, mining the complete set of frequent subsequences is time consuming for large sequence databases. Wang et al. [62] propose the use of a new CONTOUR algorithm to mine directly and efficiently a subset of high-quality subsequences and cluster the input sequences. They mainly focus on designing effective pruning techniques for search spaces to accelerate the mining process and discuss the construction of an accurate clustering algorithm on the basis of the CONTOUR result. Wang et al. conducted an extensive performance study to evaluate the efficiency and scalability of CONTOUR, as well as the accuracy of the clustering algorithm based on frequent subsequence.

Chao and Wei [72] assess the vector feature of the clickstream, which is generated by mining the closed repetitive-gapped subsequence. By considering a particular task of clickstream clustering, they improve the BIRCH algorithm. The results of a performance study on several benchmark datasets reveal that this method effectively and efficiently clusters clickstreams. As a promising future work, web pages can be generalized to reduce the dimensionality of the feature vector and enhance processing speed given the same number of sessions. Thus, web page classification should be examined, and clickstreams should be aggregated in multiple granules.

In another paper, authors discover the problem of distinguishing frequently occurring patterns, or motifs in medical datasets. They suggest a novel approach based on grammar induction that provides the approximate discovery and also they propose variable-length motifs finding in streaming data [69].

**Figure 6:**
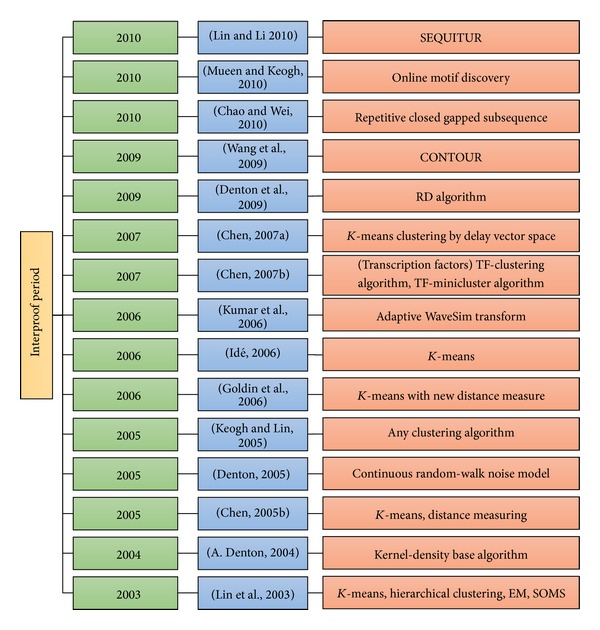
The chronology of methods in interproof period.

Mueen and Keogh [70] developed the first algorithm to discover online motifs. This algorithm monitors and maintains motifs in the most recent history of a stream in real time. This algorithm also incorporates a worst-case update time that is linear to window size and is extendible to maintain complex pattern structures. By contrast, current offline algorithms require either a significant update time or very costly preprocessing steps. The core ideas presented in this article extend the algorithm to address arbitrary data rates and to detect multidimensional motifs. Researchers have demonstrated the utility of their algorithms in a variety of case studies in the domains of robotics, acoustic monitoring, and online compression.  Table 2 and Figure 6 depict the overall review of the studies conducted during the interproof period.

### 3.3. Postproof Period (2011 to 2013)

Since 2011, studies have proposed approaches to obtain significant results for subsequence time series clustering. In the following section, we briefly describe the papers published during this period.

Rakthanmanon et al. [32] have contributed two fundamental early studies during this time. First, they explain the inherently imperfect problem in time series clustering from streams. Second, they employ the MDL framework for time series clustering; this method is efficient, productive, and parameter free. Rakthanmanon et al. [32] confirm that their method generates correct outputs with respect to the various datasets obtained from the analysis of medicine, zoology, and industrial processes. In this study, they represent clustering that can strongly ignore some of the data to group subsequences of different lengths [63].

Nunthanid et al. [73] propose a parameter-free algorithm for motif discovery called *k*-best motif discovery (kBMD). This algorithm detects *k*-best motifs without any parameters. The algorithm returns a small set of motifs, which is ranked by a proposed scoring function. The experimental results of this study demonstrate that kBMD can discover all planted patterns and that it is better than the discovery of motifs with variable lengths in terms of both the coverage of planted patterns and high accuracy-on-detection. However, this preliminary work displays a drawback; that is, the time complexity remains high because all motifs must be analyzed.

Li et al. [74] develop a methodology to discover approximate time series motifs with various lengths by using a grammar-based compression algorithm. The algorithm in this method can detect hierarchical structure, regularity, and grammar in the data. The visualization tool also enables the user to navigate different coexisting motifs with various lengths in the dataset. The results of this paper show that the grammar-based approach can determine some important motifs and that a new direction that integrates grammar-based algorithms into the discovery of time series patterns is worth exploring. Li et al. [74] also propose a search heuristic to improve the quality of induced grammar in this study. In the future, they intend to analyze the time complexity of the random search algorithm, which can be controlled by limiting the number of iterations. However, for long sequences numerous iterations may be necessary to affect the results. In the worst case, the algorithm resorts to the same grammar as SEQUITUR. Goldin et al. [64] also aim to examine and approximate the number of required iterations and the fraction of all paths that improve on base grammar. This study can enable different biases in a random search. For instance, the bias may be adjusted dynamically according to current grammar quality. Goldin et al. [64] also intend to explore other search heuristics, such as the rule ranking and filtering procedures for the visualization tool. False positives may be eliminated by estimating the distances between motif occurrences.

Another research has suggested the selective clustering of subsequence time series. The authors indicate that subsequence time series clustering can be meaningful if noise or unimportant subsequences can be ignored and if member subsequences can possess different lengths. The efficiency of the proposed algorithm is validated by testing in different data domains, such as ECG data [33]. However, this approach requires predefined constraint values that are subjective and sensitive (e.g., subsequence width).

In 2013, Madicar et al. [31] explained the process of clustering multiple time series. The clustering of subsequences within a single time series is also discussed. This research proposes a novel clustering technique without parameters. To address the lack of parameters, a discovery algorithm and some statistical principles are used to obtain the parameters. The dataset outputs confirm the efficiency of the process in selecting the appropriate subsequence width [31]. However, this process is conducted offline and is complex.

Kang et al. [71] design a new method to extract shapes from time series. This method consists of two steps: testing noise and collecting information regarding sets of features. After subsequences are extracted from time series data and are grouped into similar clusters, the noise test is conducted to check cluster accuracy. In the second step, sets of features are preserved to enhance the efficiency of the result in comparison with raw data clustering. These steps improve the result of shape patterns rather than the motif discovery algorithm proposed in [75]. An advantage of this method is that it ignores the noise from time series data and produces nonnoise subsequences, thus generating meaningful results for search machines. This method generates better results than the other methods proposed in this period with respect to noisy time series. The proposed shape extraction method can be applied to both artificial and real world data.

Yang and Wang [76] reassess the problem and suggest the phase shift weighted spherical *k*-means algorithm (PS-WSKM in short) for clustering unsynchronized time series. In PS-WSKM, the phase shift procedure is explained into the clustering process so that the phase problem is solved effectively. Meanwhile, the subsequences weights are embedded to subsequences to make the algorithm more robust.

Rakthanmanon et al. [77] suggest that, by combining four novel ideas together, they can search and mine truly massive time series for the first time. They demonstrate the following extremely unintuitive fact; in massive datasets they can exactly search under DTW much more quickly than the current Euclidean distance search algorithms.

**Figure 7:**
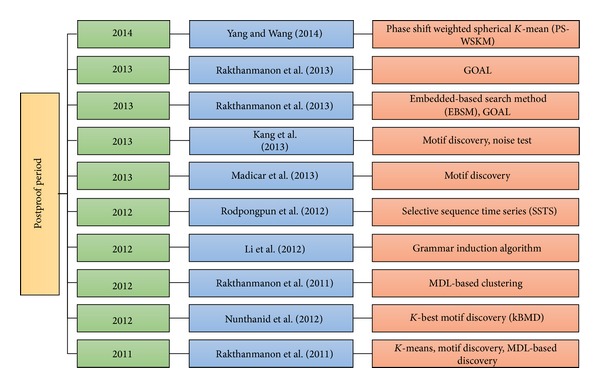
The chronology of methods in postproof period.

In the most recent article on subsequence time series clustering, Rakthanmanon et al. [57] combine four novel ideas under DTW to locate and mine large time series data. The current problem in subsequence time series clustering is searching large datasets. This issue explains why most academic work on the mining of time series data considers only a few million time series objects when billions of these objects are available for exploration in industry and science. The DTW method is faster than the Euclidean distance algorithms; hence, results can be obtained quickly. The dataset introduced in this paper is larger than the previous datasets. Rakthanmanon et al. [57] discuss how their ideas address problems in mining high-level time series data, such as motif discovery and clustering at scales that are otherwise untenable. Moreover, they efficiently support the distance measure for uniform scaling, the utility of which is underappreciated. In addition to mining large datasets reaching one trillion data points, Rakthanmanon et al. indicate that the real-time monitoring of data streams can handle faster arrival rates and/or use cheaper and lower-powered devices than other new methods.  Table 3 provides the dimensions of papers published in the postproof period, and Figure 7 chronologically clarifies the methods generated in the postproof period.

## 4. Discussion

In this section, the strengths and weaknesses of the clustering of subsequence time series are discussed. The future of subsequence time series clustering is then explained. The time series data are segmented into subsequence data, and the required details of subsequences are inputted into the similarity matrix for clustering. Subsequences are then clustered according to the similarity matrix. Finally, the clustering results are presented.

Researchers clustered time series data approximately 10 years ago but obtained insignificant results [14]. For instance, similar time series clusters are generated for each data in the end. Over time, other researchers developed solutions to this problem and proposed methods accordingly. Some techniques generated meaningful results and improved the accuracy and performance of clustering as explained in previous sections. In the following paragraphs, we evaluate the strengths and weaknesses of the studies over the three periods discussed in preceding sections.

In Table 4, strength is assessed in four different dimensions, namely, pruning the research space, using a long sequence, determining corresponding clusters, and recognizing resemblances. Weaknesses are evaluated in terms of five dimensions, that is, memory usage, unique grammar, undetected rules, limited time series, and lack of predictability. In the preproof period, researchers focused on determining corresponding clusters; some examined large subsequence clusters and they considered these clusters to be strengths in their research. However, they faced the limitations of implementing in a large-scale environment and memory usage. During this period, the ability of researchers to determine and predict appropriate groups for each cluster was weak.

The strengths and weaknesses of studies conducted in the interproof period are presented in Table 5. The research strengths at this time are assessed with respect to five dimensions, which are meaningful results, successful clustering, noise elimination, effectiveness in large window sizes, and improved BIRCH algorithm. Weaknesses are evaluated in terms of five dimensions, namely, negative view, deterministic dynamical system, large ratio, unsuccessful clustering, and limited investigation of behavior. The majority of studies focused on obtaining meaningful results in the clustering of subsequence time series because the findings of the preceding period were meaningless. Successful clustering was emphasized; however, the results of the studies did not cover a large ratio. Thus, data failed to cluster successfully. Lin et al. [27] claimed during this period that these results are meaningless. This theory viewed subsequence time series clustering negatively and discussed the cause of meaningless results in preceding papers.

Strengths' dimensions in the third period are efficient and successfulness in meaningful results, parameter-lite clustering, parameter-free clustering, and find best motif; while the weaknesses cover the complexity, not clear results, and worse result in large dimensions as presented in Table 6. Researchers have focused on finding a solution for improving the meaningless result, and they came up with a new dimension which was parameter-free clustering. Until the end of 2012, even though researchers tested experiments by parameter-free clustering, the result was not satisfactory. In 2013, Madicar et al. [31] find the algorithms for parameter-free clustering as a new solution for overcoming the meaningless result. Continually, two of the newest articles in the domain of subsequence time series clustering [57, 71] have attempted to solve the problem of unclear results and worse results in large databases. They found the noise ignoring and combining some method together under dynamic time warping can aid to handle these problems. The early studies we assessed also encountered complexity and displayed unclear results.

## 5. Conclusion

Subsequence time series clustering explores the subsequence clusters of time series data. Many studies have concentrated on related algorithms as a subroutine in rule discovery, indexing, classification, and anomaly detection. We specifically assess this clustering from the perspective of basic methods, similarity/distance measures, challenges, evaluation metrics, applications, and datasets. To summarize recent developments in this area of research, we review 25 regular articles on subsequence time series clustering. The evolution of this clustering is classified into three groups, namely, the pre-, inter-, and postproof periods. We have elucidated and compared the strengths and weaknesses of the previous literature and presented theoretical and practical issues for future study.

## Figures and Tables

**Table 1:** The overview of preproof period dimensions.

Article	Problem	Method	Algorithm	Goal	Extent
[41]	Reducing the size of the grammar and producing structure as a by-product/the input is not a continuous stream	Hierarchical clustering	SEQUITUR	Abstracting subsequences	No

[24]	Finding rules relating time series patterns	Pattern discovery	Rule finding algorithms, episode rule, simple rule discovery, *K*-means	Discovery of interesting, interpretable, and useful rules	No

[25]	Determining what distinguishes time series in that set from other time series obtained from the same source	Pattern discovery	*L*-sequences	Identifying shared patterns	No

[4]	Supervised and unsupervised learning	Pattern discovery	PERUSE	Finding recurring patterns	[25]

[26]	Determining activation and repression of specific genes	Clustering	Cluster-buster	Finding clusters of prespecified motifs in DNA sequences	No

**Table 2:** The overview of interproof period dimensions.

Article	Problem	Method	Algorithm	Goal	Extent
[27]	Meaningless time series clustering	Hierarchical and partitioning clustering	*K*-means, hierarchical clustering, EM, SOMS	Proving the claim of meaningless results	No

[45]	Specifying uninteresting sequences and their effects	Density-based clustering	Kernel-density base algorithm	Detecting meaningful pattern	[7, 12]

[78]	Sequential time series clustering is meaningless	Partitioning clustering	*K*-means, distance measuring	Showing sequential time series clustering is not meaningless	[27]

[60]	Very high noise levels	Density-based clustering	Continuous random-walk noise model	Noise elimination and high quality measure	[45]

[14]	Certain constraint in datasets and clusters, meaningless result	Hierarchical and partitioning clustering	Any clustering algorithm	Showing clustering of time series subsequences is meaningless	No

[64]	Reliable determination of the produced sequences of cluster centroids	Partitioning clustering	*K*-means with new distance measure	Results: the claim of the result of *K*-means clustering for time series subsequences is independent of the time series that created it	[14]

[79]	Sinusoidal time series clustering	Partitioning clustering	*k*-means	Explaining sine waves results of subsequence time series clustering	[14]

[5]	Hidden knowledge in time series	Hierarchical clustering, discovery pattern	Adaptive WaveSim transform	Extracting hidden knowledge in time series data	[14]

[28]	Cluster representatives are smoothed and generally do not look at all like any part of the original time series, meaningless results	Hierarchical and partitioning clustering	(Transcription factors) TF-clustering algorithm, TF-minicluster algorithm	Producing useful time series clustering	[27]

[61]	Sequential time series clustering is meaningless	Partitioning clustering	*K*-means clustering by delay vector space	Showing sequential time series clustering can indeed be meaningful	[27]

[13]	Unspecific results from dataset, meaningless	Pattern discovery	RD algorithm	Creating cluster exclusively from subsequences	[14, 60]

[62]	Time consuming to mind the complete set of frequent subsequences for large sequence databases	Pattern discovery	CONTOUR	Efficiently discovering a set of summarization subsequences	No

[72]	Categorizing visitors based on their navigation patterns on a website	Pattern discovery	Repetitive closed gapped subsequence	Constructing feature vector of click stream	[14, 61]

[70]	The detection of repeated subsequences, time series motifs	Pattern discovery	Online motif discovery	Useful extensions of the algorithm to deal with arbitrary data rates and to discover multidimensional motifs.	[75]

[69]	Identifying frequently accurate patterns or motifs	Pattern discovery	Sequitur	Discovery of approximate, variable-length motifs in streaming data.	No

**Table 3:** The summary of postproof period dimensions.

Article	Problem	Method	Algorithm	Goal	Extent
[63]	The problem of time series clustering from a single stream	Motif discovery	MDL-based clustering	Creating meaningful result	No

[32]	The problem of time series clustering from a single stream	All methods	*K*-means, motif discovery, MDL-based discovery	Producing correct results	[24]

[73]	Discovery motif with arbitrary length	Pattern discovery	*K*-best motif discovery (*k*BMD)	Developing the main idea of best motif	[80]

[74]	Length of motifs in finding time series motifs	Pattern discovery	Grammar induction algorithm	Developing a motif visualization system based on grammar induction	[81–83]

[33]	Meaningless outcomes as outputs based on inputs	Pattern discovery	Selective sequence time series (SSTS)	Achieving meaningful results	[24]

[31]	Predefined constraints values	Pattern discovery	Motif discovery	Eliminate the problem of predefined constraint values such as width of subsequences, by utilizing motif discovery algorithm	[32, 33]

[71]	Extracting and classifying shapes from very noisy real world time series	Pattern discovery	Motif discovery, noise test	A new method for shape extraction from time series	[75]

[57]	The difficulty of scaling a search to large datasets	Pattern discovery	God's algorithm (GOAL), embedded-based search method (EBSM)	Search and mine massive time series for the first time	No

[76]	Invalid subsequence time series clustering	Partitioning clustering	Phase shift weighted spherical *k*-mean (PS-WS*K*M)	Clustering unsynchronized time series	[5]

[77]	Difficulty of scaling search to large datasets	Pattern discovery	God's algorithm (GOAL)	Search and mine truly massive time series for the first time	No

**Table 4:** Strengths and weaknesses of preproof period researches.

Article	Strengths				Weaknesses				
	Pruning the research space	Using long sequence	Determining corresponding clusters	Recognizing resemblance	Memory usage	Unique grammar	Undetected rules	Limited TS	Lack of predictability
[41]		*✓*			*✓*	*✓*			

[24]	*✓*						*✓*		

[25]			*✓*					*✓*	

[4]			*✓*						*✓*

[26]				*✓*					

**Table 5:** Strengths and weaknesses of interproof period researches.

Article	Strengths					Weaknesses				
	Trying to get meaningful results	Successful clustering	Noise elimination	Effective in large window size	Improved BIRCH algorithm	Negative view	Deterministic dynamical system	Large ratio	Unsuccessful clustering	Limited investigation of behavior
[27]	*✓*					*✓*				

[12]	*✓*	*✓*					*✓*			

[60]			*✓*					*✓*		

[14]	*✓*					*✓*				

[64]	*✓*	*✓*							*✓*	*✓*

[79]	*✓*								*✓*	

[5]	*✓*	*✓*								*✓*

[28]	*✓*	*✓*								

[61]		*✓*							*✓*	*✓*

[13]		*✓*							*✓*	

[62]	*✓*	*✓*		*✓*					*✓*	*✓*

[72]					*✓*			*✓*		*✓*

[70]	*✓*							*✓*	*✓*	

**Table 6:** Strengths and weaknesses of postproof period researches.

Article	Strengths				Weaknesses		
	Efficiency and successfulness in meaningful results	Parameter-lite clustering	Parameter-free clustering	Find best motif	Complexity	Not clear results	Worse result in large dimensions
[32]	*✓*	*✓*	*✓*		*✓*	*✓*	

[73]				*✓*	*✓*		

[74]				*✓*	*✓*		*✓*

[33]	*✓*						

[31]	*✓*		*✓*		*✓*		

[71]	*✓*		*✓*	*✓*		*✓*	

[57]	*✓*		*✓*				
